# *Haemophilus parasuis* α-2,3-sialyltransferase-mediated lipooligosaccharide sialylation contributes to bacterial pathogenicity

**DOI:** 10.1080/21505594.2018.1502606

**Published:** 2018-08-12

**Authors:** Huan Wang, Lu Liu, Qi Cao, Weiting Mao, Yage Zhang, Xinyi Qu, Xuwang Cai, Yujin Lv, Huanchun Chen, Xiaojuan Xu, Xiangru Wang

**Affiliations:** a State Key Laboratory of Agricultural Microbiology, College of Veterinary Medicine, Huazhong Agricultural University, Wuhan, China; b Key Laboratory of Preventive Veterinary Medicine in Hubei Province, The Cooperative Innovation Center for Sustainable Pig Production, Wuhan, China; c Key Laboratory of Development of Veterinary Diagnostic Products, Ministry of Agriculture of the People’s Republic of China, Wuhan, China; d College of Veterinary Medicine, Henan University of Animal Husbandry and Economy, Zhengzhou, China

**Keywords:** α-2, 3-sialyltransferase, *lsgB*, LOS sialylation, adhesion and invasion, serum resistance, pathogenicity

## Abstract

Bacterial lipooligosaccharide (LOS) is an important virulence-associated factor, and its sialylation largely confers its ability to mediate cell adhesion, invasion, inflammation, and immune evasion. Here, we investigated the function of the *Haemophilus parasuis* α-2,3-sialyltransferase gene, *lsgB*, which determines the terminal sialylation of LOS, by generating a *lsgB* deletion mutant as well as a complementation strain. Our data indicate a direct effect of *lsgB* on LOS sialylation and reveal important roles of *lsgB* in promoting the pathogenicity of *H. parasuis*, including adhesion to and invasion of porcine cells *in vitro*, bacterial load and survival *in vivo*, as well as a contribution to serum resistance. These observations highlight the function of *lsgB* in mediating LOS sialylation and more importantly its role in *H. parasuis* infection. These findings provide a more profound understanding of the pathogenic mechanism of this disease-causing bacterium.

## Introduction

*Haemophilus parasuis* is a small, non-*motile*, pleomorphic rod-shaped, and nicotinamide adenine dinucleotide (NAD)-dependent bacterium in the *Haemophilus* genus of the family *Pasteurellaceae* [1]. As a commensal pathogen, *H. parasuis* colonizes the upper respiratory tract of healthy pigs and forms a symbiotic relationship with the host [1]. When the host suffers from environmental stresses or reduced immunity, it can spread to the lungs and cause pneumonia or even get into the blood circulation and invade multiple tissues to cause systemic Glässer’s disease, characterized by fibrinous polyserositis, polyarthritis and meningitis in pigs [2–4].

To date, 15 serotype strains of *H. parasuis* with diverse virulence have been described, but the virulence factors of this bacterium are not completely defined. Early studies described a neuraminidase activity located in the outer membrane of *H. parasuis* [5,6]. This indicates a new antigenic epitope of *H. parasuis*, but the detailed biological function of sialylation in this bacterium is unknown. Sialylation is formed by specific cellular sialyltransferase-mediated transfer of sialic acids to carbohydrate chains [7]. The sialyltransferases catalyze the transfer of a sialic acid residue from a cytidine monophosphate (CMP)-activated donor substrate, typically cytidine monophosphate-N-acetylneuraminic acid (CMP-Neu5Ac), onto a carbohydrate receptor, which is usually a galactose residue, or N-acetyl residue (GalNAc) or other sialic acid [8]. Sialylated lipooligosaccharide (LOS) is widely present in bacteria, including *Neisseria gonorrhoeae, Campylobacter, Pasteurella* and Luminescent Bacteria [9–12]. Sialylation is not only involved in the stability of protein structure, but also in extension of the half-life of glycoproteins; moreover, it is an important component in many biological processes, such as cell recognition [13], leukocyte homing [14], cell adhesion and invasion [15] and cell signaling [16]. The genes involved in sialic acid metabolism have been investigated in 21 *H. parasuis* strains from different clinical origins (including nasal and systemic isolates). These genes include the neuraminidase gene *nanH*, the CMPNeu5Ac synthetase, and the sialyltransferase genes *neuA, siaB* and *lsgB* [17]. Commonly, the sialyltransferase has been classified into α-2,3-sialyltransferase, α-2,6-sialyltransferase and α-2,8-sialyltransferase [18]. Based on their protein sequence homology, all the sialyltransferases identified to date could be divided into six glycosyltransferase (GT) families GT4, GT29, GT38, GT42, GT52 and GT80 according to the Carbohydrate-Active enZymes (CAZy) (http://www.cazy.org/) database [19]. Noticeably, all sialyltransferases from eukaryotes and some viruses are grouped into GT29 families, while bacterial sialyltransferases are grouped into GT4, GT38, GT42, GT52 and GT80 families [18]. The α-2,3-sialyltransferase encoded by *H. parasuis lsgB* gene has been grouped into GT38 family [20]. This *lsgB* gene was predominantly present in the systemic *H. parasuis* isolates but was not present in any of the nasal isolates [17], suggesting that *lsgB*-mediated sialylation is likely to affect bacterial pathogenicity. However, the influence of sialylation in *H. parasuis* pathogenicity is not clear and its mechanism of action remains to be determined.

In this study, we investigated the function of the α-2,3-sialyltransferase gene, *lsgB*, in *H. parasuis* serotype 5 virulent strain SH0165 by generating a *lsgB* deletion mutant and its complementation strain. We provide evidence that *H. parasuis lsgB* plays an important role in LOS terminal sialylation, bacterial pathogenicity, adhesion and invasion, and resistance against the complement alternative pathway-mediated bactericidal effect in the serum. Characterization of sialylation in *H. parasuis* expands understanding of bacterial infection and contributes to the elucidation of *H. parasuis* pathogenic mechanisms.

## Results

### *H. parasuis* strain SH0165 contains the conserved motifs of the sialyltransferase LsgB

**Figure 1.**
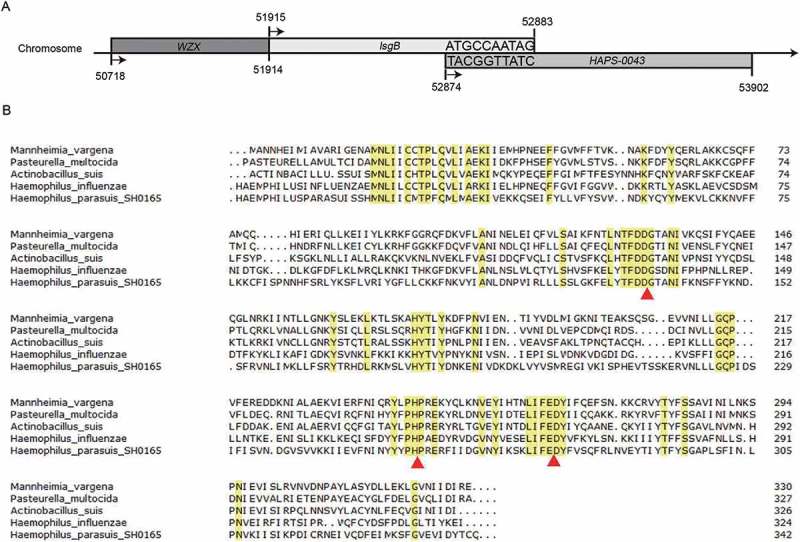
Alignment of the LsgB protein among *H. parasuis* and other genera. (a) Simplified depiction of the *lsgB* gene and its flanking genes on the *H. parasuis* SH0165 chromosome. (b) Analysis of LsgB protein homology among different bacterial genera, including *M. vargena, P. multocida, A. suis, H. influenzae* and *H. parasuis* using the NCBI BLAST program with the default parameters. Conserved protein sequences are labeled in yellow, and the triangles indicate the conserved motifs of the LsgB sequence.

To clarify the *lsgB* region available for deletion, we analyzed the *lsgB* gene locus and its flanking genes published in the SH0165 genomic sequence on the NCBI website. We found that *lsgB* has 10 bases that overlap with its downstream gene, HAPS-0043 (Figure 1(a)). Bacterial sialyltransferases usually contain two conserved motifs, the aspartic acid/glutamic acid-aspartic acid/glutamic acid-glycine motif (E/D-E/D-G), and the histidine-proline (H-P) motif, which are involved in substrate binding and catalytic activity [21]. We compared LsgB protein sequences in multiple bacterial genera, including *Mannheimia vargena, Pasteurella multocida, Actinobacillus suis, Haemophilus influenzae* and *H. parasuis*, and found that the conserved LsgB motifs, marked with red triangles in Figure 1(b), were present in *H. parasuis* SH0165, as well as in the other bacteria. Thus, an in-frame gene deletion was engineered in the region containing these conserved motifs.

### PCR identification of the *ΔlsgB* mutant and its complementation strain C-lsgB

**Figure 2.**
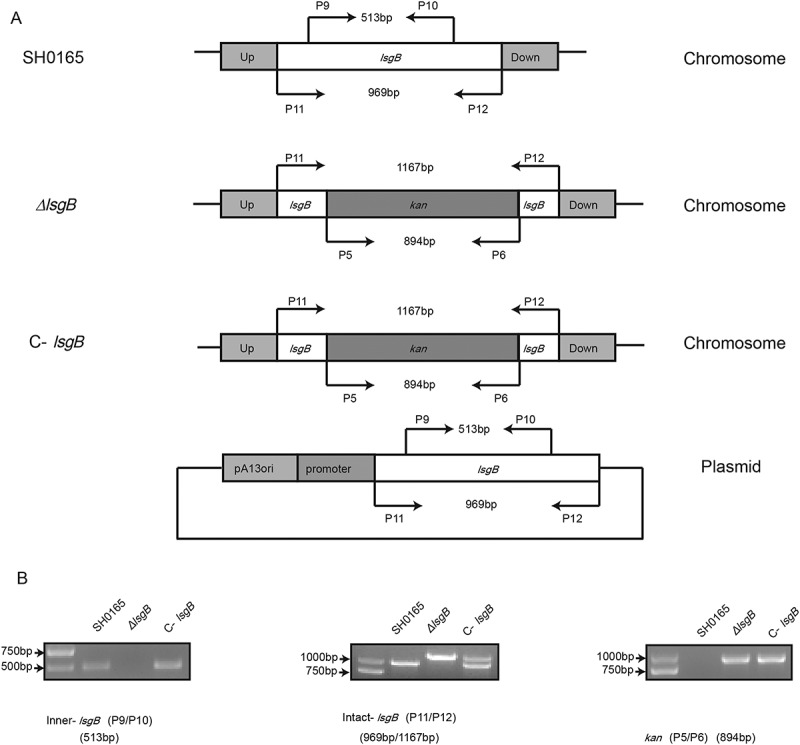
Schematic representation and PCR identification of the *lsgB* gene deletion and its complementation. (a) The primers for amplification of specific target fragments are labeled. Primer pair P9/P10 was used to detect the inner *lsgB* gene, P11/P12 was used to amplify the intact *lsgB* deletion region, and P5/P6 amplified the kanamycin cassette. (b) PCR identification of the inner-*lsgB* region (513 bp), the intact deletion region of *lsgB* (969 bp or 1167 bp), and the kanamycin resistance cassette (894 bp).

The *lsgB* deletion mutant was constructed in wild-type SH0165 by natural transformation, and the complementation strain, C-*lsgB*, was generated by introducing the whole *lsgB* operon in the shuttle vector pSHK3-Gm as described in Materials and Methods. As shown in Figure 2(a), two primer pairs, P9/P10 and P11/P12, were used to detect internal and intact *lsgB* fragments, respectively, and the primer pair P5/P6 was used to detect the kanamycin fragment. In the *ΔlsgB* mutant, P9/P10 cannot amplify the inner *lsgB* product, and the C-*lsgB* strain yielded the same band as the wild-type strain (513 bp) (Figure 2(b)). When amplified with P11/P12, the *ΔlsgB* mutant yielded a fragment (1167 bp) that was a little bigger than that from the wild-type (969 bp), indicating successful replacement of the internal *lsgB* fragment by the kanamycin cassette. As expected, amplification with P11/P12 in the C-*lsgB* strain generated two bands that were exactly the same as those from both wild-type and the *ΔlsgB* strains, indicating successful complementation of the mutant *lsgB* gene in the C-*lsgB* strain (Figure 2(b)). The kanamycin cassette (894 bp) was detected in both *ΔlsgB* and C-*lsgB* strains, but not in the wild-type strain (Figure 2(b)). Together, these results indicate successful construction of the *lsgB* deletion mutant and its complementation strain.

### Deletion of the *lsgB* gene in *H. parasuis* leads to elongated rod shape morphology and terminal desialylation of LOS

**Figure 3.**
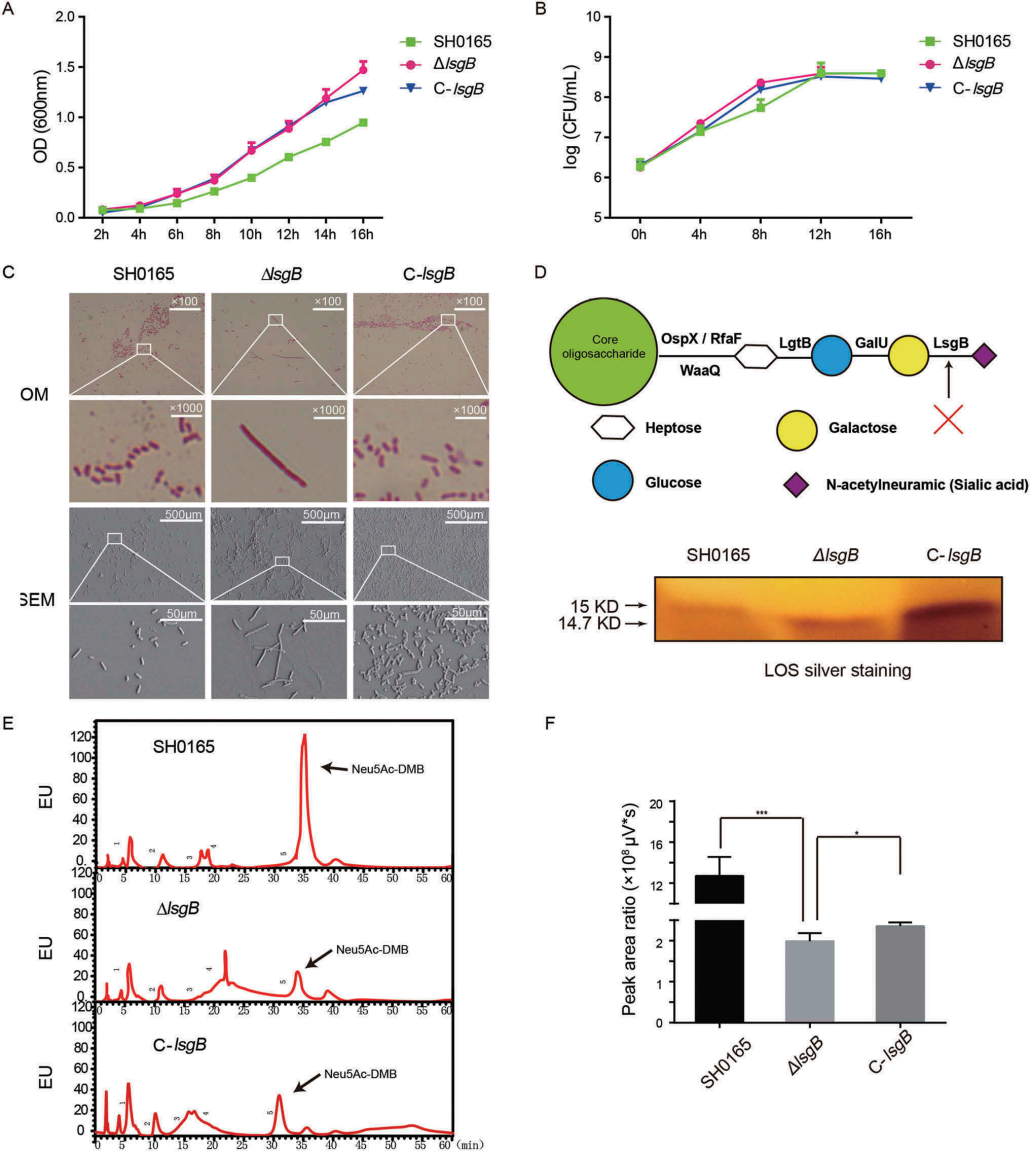
Effects of *lsgB* deletion on bacterial growth, morphology, and LOS sialylation. (a-b) Comparison of growth among the wild-type SH0165, *ΔlsgB* mutant and C-*lsgB* strains. OD_600_ values of each strain during growth for 16 h were recorded every 2 h, and viable bacterial counts were calculated at 4 h intervals. (c) Both optical microscopy and scanning electron microscopy were used to observe the morphology of each strain. (d) Schematic depiction of the LOS structure and silver staining of LOS in the wild-type SH0165, *ΔlsgB* mutant and C-*lsgB* strains. (e-f) HPLC analysis of LOS sialic acid content in the SH0165, *ΔlsgB* and C-*lsgB* strains. The target peak was detected by fluorescence at appropriate time points based on the results from a standard substance (e), and the peak area ratios were compared (f).

We first compared the growth of wild-type SH0165, *ΔlsgB* mutant and C-*lsgB* strains. As shown in Figure 3(a,b), the OD_600_ values of the *ΔlsgB* mutant and C-*lsgB* cultures increased slightly faster than that of wild-type SH0165, but the viable bacterial counts during 16 h of growth were not significantly different among strains. We next compared morphology of the bacteria by Gram-staining and scanning electron microscopy and found that deletion of *lsgB* led to an elongated rod shape, while the C-*lsgB* strain exhibited a similar morphology as the wild-type strain (Figure 3(c)).

*lsgB* determines the terminal sialylation of LOS (Figure 3(d)); therefore, we next extracted the LOS from each strain and compared potential differences via SDS-PAGE with silver staining. We observed a slight difference in the molecular weight of LOS, which was around 14.7 kDa in the *ΔlsgB* mutant, but slightly larger in both the wild-type and C-*lsgB* strains (around 15 kDa) (Figure 3(d)). This observation indicated differences in sialic acid content among these strains. Subsequently, HPLC was applied to quantitatively analyze the difference of sialic acid content. The results showed that total sialic acid content in the *ΔlsgB* mutant was significantly lower than that in the wild-type strain, while *lsgB* complementation could significantly increase the sialic acid content in the *ΔlsgB* mutant, although it was still much lower than the wild-type SH0165 (Figure 3(e,f)). These observations revealed an important role of *lsgB* in maintaining the sialylation of LOS, and deletion of *lsgB* led to a molecular weight reduction and the desialylation of LOS.

### *lsgb* deletion in *H. parasuis* attenuates bacterial autoagglutination, pathogenicity, and in vivo colonization

**Figure 4.**
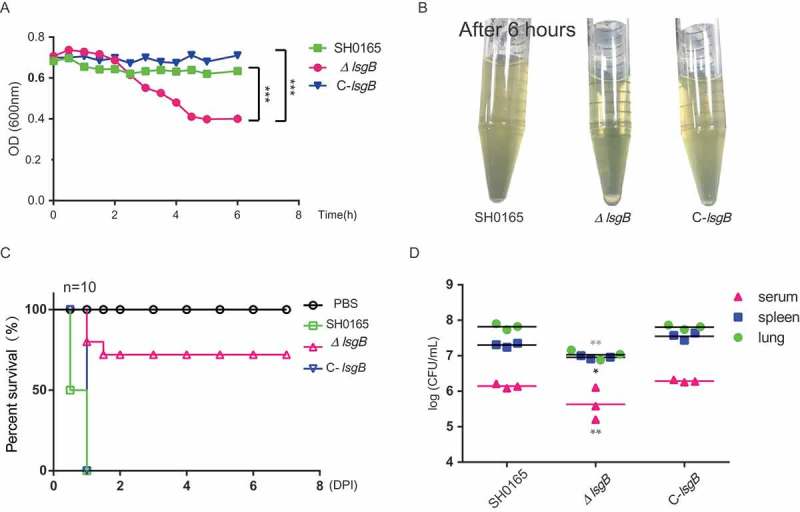
*H. parasuis lsgB* deletion attenuates bacterial autoagglutination and pathogenicity, and colonization and survival *in vivo*. (a-b) OD_600_ values of the upper suspension of each strain were measured and compared every 30 min (a), and the culture suspension after 6 h of static incubation was photographed (b). (c) Virulence test of the wild-type SH0165, *ΔlsgB* mutant and C-*lsgB* strains by comparing survival of mice post infection. Each group contained 10 mice, and survival was monitored for 7 days. (d) Bacterial colonization in spleen and lung and survival in the blood were compared in an 8 h challenge assay. Bacterial counts were determined by serial dilution and plating and are expressed as logCFU/mL.

Bacterial autoagglutination is a potential virulence-associated indicator in some Gram-negative bacteria [22]; therefore, we next considered whether *lsgB* gene deletion affects bacterial virulence. In the autoagglutination test, the upper suspension of the static *ΔlsgB* culture rapidly decreased with time (Figure 4(a)) and most bacteria had precipitated to the bottom of the culture after incubation for 6 h (Figure 4(b)), indicating significantly attenuated autoagglutination of the *ΔlsgB* mutant compared with the wild-type and C-*lsgB* strains.

Moreover, these strains were intraperitoneally injected into mice and their *in vivo* pathogenicity was analyzed by monitoring the survival rates of the mice. After 12 h post challenge, only 50% of the mice challenged by the wild-type strain had survived, and all mice in both wild-type and the C-*lsgB* groups died within one day post infection. However, mice in the *ΔlsgB* challenge group had a survival rate of 75% at the end of the observation period. Mice receiving PBS injection were alive throughout the experiment (Figure 4(c)). We also compared the *in vivo* bacterial load of the wild-type SH0165, *ΔlsgB* mutant, and C-*lsgB* strains in mice in an 8 h infection assay. The bacterial load of the *ΔlsgB* mutant in the spleen and lung and its survival in serum was significantly reduced compared with that of the wild-type strain. The recovered C-*lsgB* strain exhibited similar level as the wild-type strain (Figure 4(d)). These results suggest that deletion of *lsgB* in *H. parasuis* largely attenuated its pathogenicity.

### Desialylation in the *ΔlsgB* mutant enhances bacterial adhesion by exposure of its galactose residues

**Figure 5.**
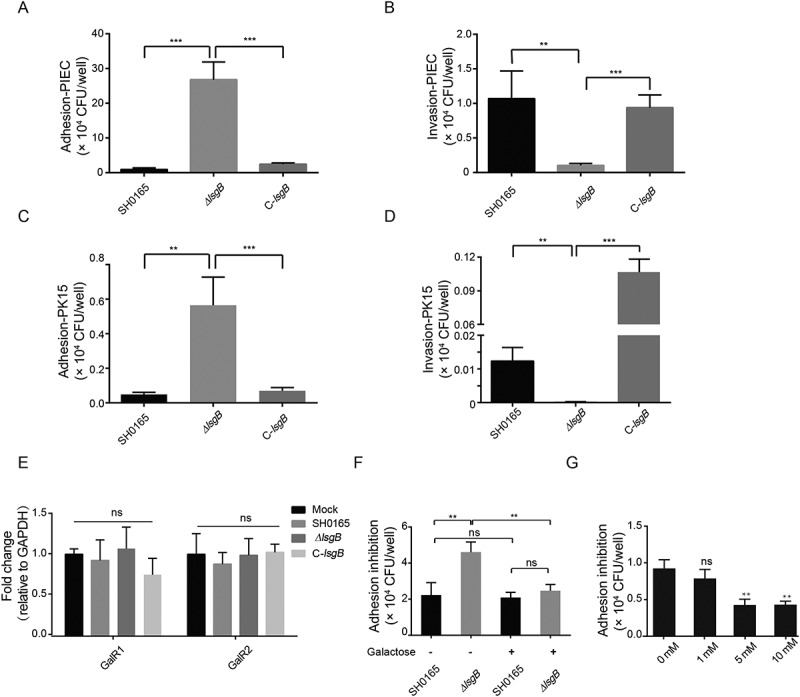
Effects of *lsgB* deletion in *H. parasuis* on bacterial adhesion to and invasion of porcine cells. (a-b) Adhesion to and invasion of PIECs by the wild-type SH0165, *ΔlsgB* mutant and C-*lsgB* strains. (c-d) Adhesion to and invasion of PK15 cells by the wild-type SH0165, *ΔlsgB* mutant and C-*lsgB* strains. (e) Real-time PCR detection of galactose binding receptor, GalR1 and GalR2, expression in response to the three strains. GAPDH was used as the endogenous control. (f) Elevated adhesion to cells by the *ΔlsgB* mutant was significantly blocked by the addition of galactose. (g) Galactose addition resulted in a dose-dependent inhibition of adhesion by the *ΔlsgB* mutant.

We have demonstrated *in vivo* attenuated pathogenicity of the *ΔlsgB* mutant compared to the wild-type and complementation strains. Therefore, we next investigated the *in vitro* adhesion and invasion abilities of these strains on PK15 and PIEC cells. As shown in Figure 5(a-d), the invasion of the *ΔlsgB* mutant in both cell types was significantly lower than that of the wild-type and C-*lsgB* strains (Figure 5(b,d)), while unexpectedly, adhesion to both cell types by the *ΔlsgB* mutant was significantly higher than that of the wild-type and C-*lsgB* strains (Figure 5(a,c)). Since *lsgB* deletion led to LOS terminal desialylation as well as the galactose residues exposure, we next determined the transcriptional change of the cellular galactose receptors GalR1 and GalR2 in response to these strains. We did not observe the differential expression of these two receptors in response to the wild-type SH0165, *ΔlsgB* mutant, and the C-*lsgB* strain (Figure 5(e)), however, when cells were pretreated with galactose, the high level of adhesion by *ΔlsgB* mutant was significantly decreased, showing a similar level as that of the wild-type SH0165 (Figure 5(f)), indicating that addition of galactose might block the galactose receptors, thus preventing the LOS terminal galactose-mediated bacterial adhesion. Moreover, we showed dose-dependent blocking of *ΔlsgB* mutant adhesion by the addition of galactose (Figure 5(g)), further supporting our hypothesis that *lsgB* deletion in *H. parasuis* results in exposure of galactose, which promotes galactose-mediated bacterial adhesion to the cells. These observations indicate that *lsgB* deletion enhances galactose exposure and galactose-mediated adhesion to cells.

### *H. parasuis lsgB* deletion attenuates resistance to complement system-mediated killing via decreased binding of the complement inhibitor fH and increased deposition of C3b and MAC

**Figure 6.**
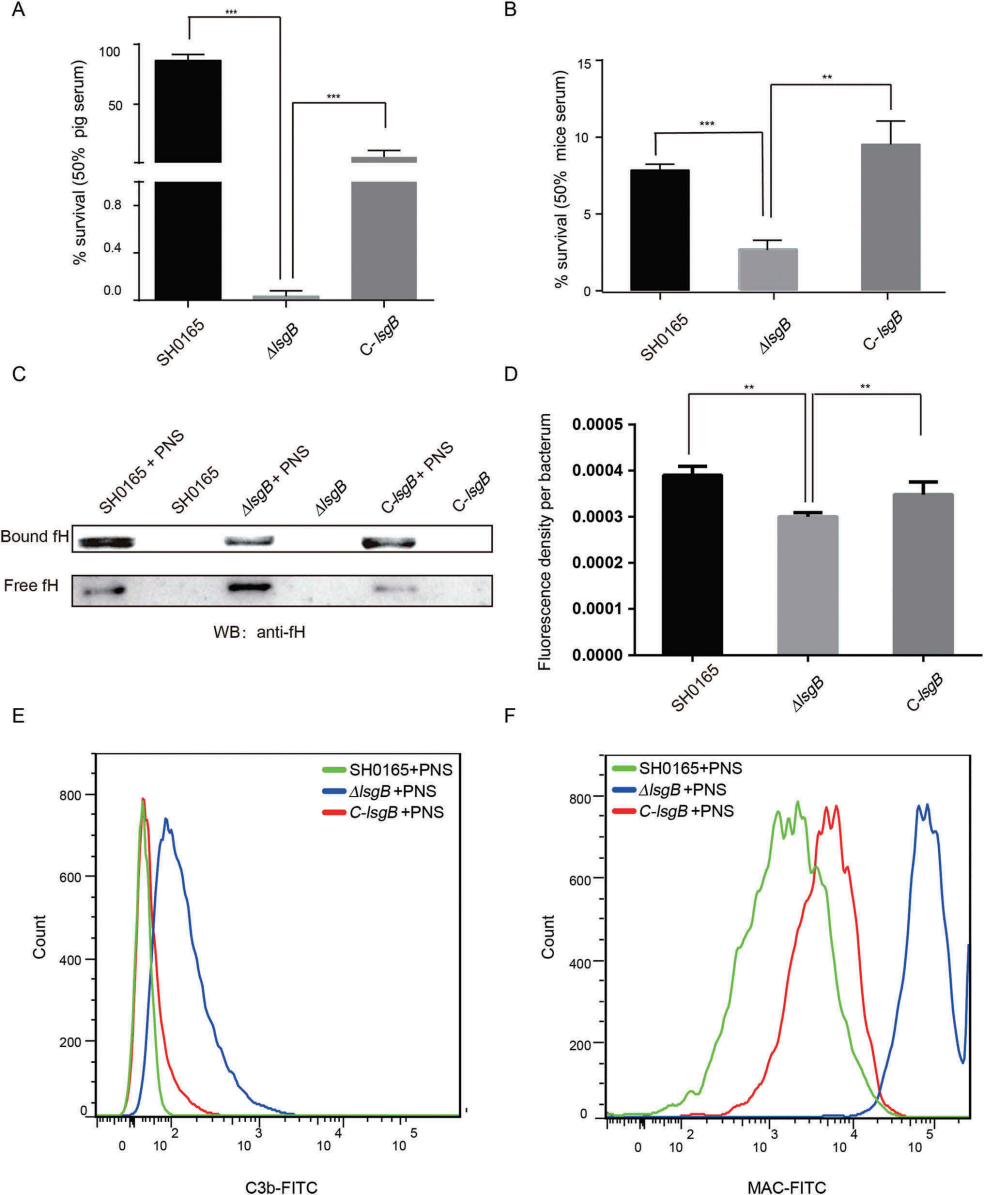
*lsgB* deletion increases bacterial sensitivity to serum complement-mediated killing. (a-b) Survival of the wild-type SH0165, *ΔlsgB* mutant and C-*lsgB* strains in response to 50% pig serum (a) and 50% mice serum (b). (c) Western blotting analyzing the recruitment of the complement inhibitor, fH, to each strain incubated with or without porcine normal serum (PNS). Both free fH (lower) and bound fH (upper) were all detected. (d) Whole cell ELISA was applied to analyze the bound fH on each strain. The results are presented as the average fluorescence density per bacterium. (e-f) Comparison of C3b or MAC deposition on the surface of the wild-type, *ΔlsgB* mutant and C-*lsgB* strains after incubation with PNS and flow cytometry.

We observed in the mouse infection assay that the *ΔlsgB* mutant showed attenuated pathogenicity and decreased *in vivo* colonization ability, including reduced survival in serum (Figure 4). We therefore questioned whether these attenuated abilities of the *ΔlsgB* mutant are associated with bacterial survival in the blood. Serum resistance assays with 50% porcine or mouse serum showed that the *ΔlsgB* mutant was much more sensitive to both sera, with the survival rate being significantly lower than those of the wild-type SH0165 and C-*lsgB* strains (Figure 6(a,b)). Moreover, these strains were incubated with or without porcine normal serum (PNS) for 1 h and their abilities of bind fH were investigated by western blotting and whole cell ELISA. After incubation, we observed that the amount of SH0165-bound fH was significantly higher than that of free fH in the supernatant. In contrast, the amount of *ΔlsgB*-bound fH was much lower than that of the free fH, and of SH0165-bound fH. The C-*lsgB* strain exhibited the same trend as the wild-type strain, showing a higher level of bound fH (Figure 6(c)). This result was further supported by the whole cell ELISA assay showing that bound-fH fluorescence density per bacterium in the *ΔlsgB* mutant was significantly lower than that in either the wild-type strain or the C-*lsgB* strain (Figure 6(d)). Complement-mediated killing relies on the deposition of C3b and formation of the MAC; therefore, we next used flow cytometry to investigate the surface deposition of these two components on the bacteria. Compared with the wild-type strain, the deposition of C3b on the *ΔlsgB* mutant was significantly increased after incubation with porcine normal serum for 30 min. The C-*lsgB* strain exhibited slightly more C3b deposition than the wild-type strain, but still less than the *ΔlsgB* mutant (Figure 6(e)). The deposition of MAC on these strains was similar to that of C3b, with a significantly increased deposition of MAC on the *ΔlsgB* mutant (Figure 6(f)). Together, these results reveal that *lsgB* deletion in *H. parasuis* leads to decreased fH binding and increased deposition of C3b and MAC on the bacteria, resulting in higher sensitivity to the serum complement system.

## Discussion

*H. parasuis* is recognized worldwide as one of the most important bacterial pathogens in the pig industry and is the leading cause of mortality during porcine weaning and nursery stages [23]. As an important opportunistic pathogen, it normally colonizes the upper respiratory tract of healthy pigs without causing any clinical symptoms as well as inducing the production of specific antibodies [24]. However, the disease is always easily to be observed under certain stress conditions especially when piglets are experiencing the immunocompromised status. For examples, maternal antibody reduction is a critical factor that allows the development of *H. parasuis* infection. Sow-reared piglets are usually protected against early infection of *H. parasuis via* acquisition of the maternal antibody [25], whereas the colostrums-deprived piglets are extremely susceptible to the systemic infection [26]. Selective pressure from the antibiotics sometimes induces the disease by the emergence of antimicrobial-resistance strains and the diversity of the isolates, thereby facilitating the distribution of *H. parasuis* [27]. Additionally, things will get worse in piglets co-infected with some other respiratory pathogens or immunosuppressive pathogens, for example, swine influenza virus could aggravate the lesions to the lung and thus facilitates microbial replication in the lung [28]. Despite of these external causes, *H. parasuis* also utilizes its internal factors for initial colonization, probably *via* adhesion to and invasion of host epithelial cells [29–31], disrupting mucosal immunity by degradation of IgA [32], resisting against phagocytosis by macrophage as well as bactericidal effect by the complement system [33–35]. Therefore, identification and characterization of the critical virulence factors that participating in these important phenotypes should provide better understanding of *H. parasuis* pathogenicity. However, only a few virulence factors in this bacterium have been truly reported to date, and the knowledge regarding the pathogenesis of *H. parasuis* still needs expanding.

Sialic acid, a carbohydrate residue at the terminus of LPS in most Gram-negative bacteria, is one of the first molecules to directly interact with host cells during bacteria-host interactions [36]. Many pathogenic bacteria, such as *H. influenzae, N. gonorrhoeae*, and *N. meningitides*, can decorate their LOS by sialylation, either by incorporating Neu5Ac into their LOS, cell membrane or capsule. Alternatively, some of the *Escherichia coli* strains and *N. meningitidis* produce capsular polysaccharides containing Neu5Ac [37]. The mechanism of LOS sialylation in these microbes differs. For example, *N. gonorrhoeae* cannot synthesize Neu5Ac but utilizes a surface α-2, 3-sialylatransferase to transfer Neu5Ac from blood circulatory CMP-Neu5Ac to the terminal galactose residue of LOS [38,39]. In *H. parasuis*, the *lsgB* gene product, α-2,3-sialyltransferase, sialylates the terminal galactose of LOS by using 5ʹ-cytidinemonophospho-N-acetylneuraminic acid or other donors [20]. Moreover, *H. influenzae* sialylates its LOS by a precursor scavenging mechanism [40], and an analogous strategy has also been reported in *H. ducreyi* [41] and *H. somnus* [42]. These observations indicate that LOS sialylation is an important modification in members of the *Haemophilus* genus.

To reveal the biological function of sialic acid in LOS modification in *H. parasuis*, we constructed an α-2,3-sialyltransferase *lsgB* gene deletion mutant, and its complementation strain. The basic characteristics of these strains were compared, including growth, morphology, and LOS sialylation. The viable bacterial counts among these strains remained the same during 16 h of culture, but the OD_600_ values of the *ΔlsgB* mutant and C-*lsgB* strains increased faster than that of the wild-type strain. We assumed that this difference might result from an elongated shape of the *ΔlsgB* bacteria, which was demonstrated by optical microscopy and SEM. However, this assumption does not apply to the complementation strain because the rod-shape of these cells was not elongated, indicating that the *lsgB* gene complementation might not fully revert all wild-type phenotypes. On one hand, the alteration of some phenotypes resulting from gene deletion might be irreversible; and on the other hand, the higher abundance of the *lsgB* gene in the C-*lsgB* strain might account for inconsistencies between the wild-type and complementation strains. Moreover, we investigated specific differences in LOS from these three strains and found that *lsgB* deletion in *H. parasuis* caused a molecular weight reduction of LOS, and correspondingly LOS sialylation in the *ΔlsgB* mutant was significantly lower than in the wild-type and C-*lsgB* strains. This reveals that the *lsgB* gene is essential for LOS sialylation in *H. parasuis*.

Self-autoagglutination is an indirect indicator of bacterial colonization and virulence *in vivo* [22,43]. Here, we observed that the autoagglutination ability of the *ΔlsgB* mutant was significantly decreased, and we also demonstrated a significant attenuation of *in vivo* pathogenicity, as well as reduced colonization and survival of the *ΔlsgB* mutant, compared to the wild-type and complementation strains. To further dissect these differences, we subsequently investigated *in vitro* adhesion and invasion abilities on two types of porcine cell, PIEC and PK15 cells. LOS is important in host cell adhesion and invasion, which is an important mechanism for *H. parasuis* infection [30]. Also, the *lsgB* gene is reported to be present predominantly in systemic clinical isolates but not in nasal isolates [17], indicating a potential relationship in *H. parasuis* strains between the *lsgB* gene and adhesion or invasion. The *ΔlsgB* mutant exhibited significantly attenuated invasion of both PIEC and PK15 cells; however, unexpectedly; we observed a prominent and significant increase in the adhesion of the *ΔlsgB* mutant to both cell types. Deletion of *lsgB* led to the exposure of the penultimate galactose of LOS; therefore, we tested whether these cell types have differences in the expression of the galactose binding domains or receptors, such as the galactose binding lectin domain (GalR) of the adhesion G protein-coupled receptor [44,45], but the results did not suggest any difference on the expression of GalR1 or GalR2 in response to these strains. However notably, our adhesion inhibition assays showed that addition of galactose significantly blocked adhesion of the *ΔlsgB* mutant in a dose-dependent manner, while adhesion of the wild-type strain was completely unaffected, revealing that it is the terminal galactose residue, which is exposed after the removal of sialic acid by *lsgB* deletion (see Figure 3(d)), that mediates the high level of adhesion.

We also compared the serum survival of the wild-type, *ΔlsgB* mutant, and C-*lsgB* strains, and found that deletion of *lsgB*, which results in desialylation of LOS in *H. parasuis*, significantly attenuated bacterial resistance against serum-mediated killing. Bacterial serum resistance is also recognized to be an important virulence indicator by assisting with bacterial survival in host blood. A previous study has also reported the contribution of LOS sialylation to serum resistance in *N. gonorrhoeae* [46]. This sialylation of *N. gonorrhoeae* LOS converts serum-sensitive strains to serum resistant strains and requires the exogenous addition of CMP-Neu5Ac and has been termed “unstable serum resistance” [47]. Several hypotheses concerning the mechanism of this “unstable serum resistance” have been raised, such as decreased binding of IgG, or increased binding of host complement alternative pathway inhibitor, fH [48]. Here, we observed that the binding of fH by *ΔlsgB* mutant was significantly less than that by both wild-type SH0165 and C-*lsgB* strains, indicating that the *H. parasuis lsgB* gene can facilitate bacterial binding of fH in response to serum challenge. Together with the attenuated inhibition of the host complement system, we also observed more deposition of C3b and MAC on the surface of the *ΔlsgB* mutant. C3b and MAC are key players in the activation of complement-mediated killing; therefore, the desialylation of LOS, because of *lsgB* deletion, resulted in a higher activation of the host complement system, and an enhanced bactericidal effect. There is an additional possibility that sialylated LOS masks epitopes that are usually recognized by pentameric IgM and that restrict the recognition of C1q and the following activation of the classical pathway in the complement system [49]. Whether LOS desialylation following *lsgB* deletion partially facilitates the engagement of IgM with certain surface epitopes in *H. parasuis*, thus boosting the classical complement pathway-mediated killing, should be further investigated.

In summary, we characterized the function of the *lsgB* gene in *H. parasuis* by generating its deletion mutant and the complementation strain. We provide evidence supporting the involvement of *lsgB* in LOS terminal sialylation, bacterial autoagglutination, and pathogenicity *in vivo* and *in vitro*. Strikingly, we found the *lsgB* deletion significantly increased bacterial adhesion to host cells by enhancing exposure of the galactose residue and its interaction with cellular galactose binding domains. Furthermore, the *ΔlsgB* mutant attenuated its binding with fH and recruited the deposition of C3b and MAC, thereby activating complement-mediated elimination of the bacteria. Characterization of *lsgB*-mediated LOS sialylation in *H. parasuis* should deepen our understanding of *lsgB* gene function and help to reveal the pathogenic mechanism of infection by this bacterium.

## Materials and methods

### Bacterial strains, plasmids and cell culture conditions

The bacterial strains and plasmids used in this study are listed in Table 1. All plasmids were propagated in *Escherichia coli* DH5α growing in Luria-Bertani medium (Oxoid, Hampshire, UK) at 37°C. *H. parasuis* clinical isolate SH0165 and its derivatives were cultured on tryptic soy agar (TSA) or in tryptic soy broth (TSB) (Difco Laboratories, Michigan, USA) supplemented with 10 μg· mL^−1^ NAD (Sigma-Aldrich, Missouri, USA) and 5% (v/v) inactivated bovine serum at 37°C (TSA/V/S, TSB/V/S, respectively). For selection of the plasmid-containing strains, the culture medium was supplemented with kanamycin at 50 μg· mL^−1^ (Kan^50^) or gentamicin at 20 μg· mL^−1^ (Gen^20^). Porcine kidney cells (PK15) and porcine iliac artery endothelial cells (PIEC) were cultured in Dulbecco’s modified Eagle’s medium (Invitrogen, Carlsbad, CA, USA) with 10% heat-inactivated fetal bovine serum in a 37°C incubator under 5% CO_2_ until monolayer confluence.

**Table 1.** Bacterial strains and plasmids used in this study.

Strains and plasmids	Characteristics	Source
*E. coli* DH5α	F–ϕ80 lacZΔM15Δ (lacZYA-argF) U169 *endA1 recA1 hsdR17* (rk-, mk+) *supE44λ-thi-1 gyrA96 relA1 phoA*	Transgen Biotech, Beijing, China
*H. parasuis* SH0165 (WT)	*H. parasuis* serotype 5 field isolate	This study
SH0165*ΔlsgB::kan* (*ΔlsgB*)	*lsgB* mutant of *H. parasuis* SH0165, Kan resistance	This study
SH0165-C-*lsgB* (C-*lsgB*)	The complement of *H. parasuis* SH0165*ΔlsgB::kan* containing pSHK3-*C-lsgB*, Kan resistance, Gm resistance	This study
pK18mobsacB	Suicide and narrow-broad-host vector, Kan resistance	[42]
pK18-*lsgB*-UKD	A 1965-bp overlap fragment containing Kan, the upstream and downstream sequences of the *lsgB* gene in pK18mobsacB, Kan resistance	This study
pSHK3	*E. coli – H. parasuis* shuttle vector, Kan resistance	[43]
pSHK3-Gm	Kan is replaced by Gm (534 bp) in pSHK3, Gm resistance	[2]
pSHK3-C-*lsgB*	A fragment containing the 769-bp promoter and complete *lsgB* ORF in pSHK3-Gm, Gm resistance	This study

### Alignment of *LsgB* protein sequences

Based on available sequence information of *H. parasuis* SH0165, the *lsgB* gene encodes an α-2,3-sialyltransferase, which belongs to glycosyltransferase family 52 and modifies bacterial LOS by sialylation. Bacterial sialyltransferases in different CAZy families (GT-38 and GT-52) and pfam family 05855 usually contain two short motifs (D/E-D/E-G and HP) [21]. The LsgB protein sequence of *H. parasuis* SH0165 was therefore compared with sequences in other congenetic Gram-negative bacteria, such as *M. vargena, P. multocida, A. suis*, and *H. influenzae*, to analyze protein homology and to identify possible conserved motifs using the National Center for Biotechnology Information (NCBI) Basic Local Alignment Search Tool (BLAST) program with the default settings [50].

### Construction of an *lsgB* deletion mutant and the complementation strain

Oligonucleotide primers used to engineer an *lsgB* deletion mutant and its complementation strain are listed in Table 2. The 546-bp upstream and 527-bp downstream fragments flanking *lsgB* were amplified from the SH0165 genome using primer pairs P1/P2 and P3/P4, respectively. Both sets of primers contained a 9-bp core DNA Uptake Signal Sequence (USS) of 5ʹ-ACCGCTTGT-3ʹ [51]. The kanamycin resistance cassette (894 bp) was amplified from pSHK3 with primers P5/P6. These three fragments were then fused by overlapping extension PCR using primers P1/P4. The resulting product was cloned into pK18mobsacB at *Bam*HI and *Pst*I sites to obtain the recombinant plasmid, pK18-*lsgB*-UKD. This was introduced into *H. parasuis* strain SH0165 by natural transformation as previously described with slight modifications [52]. Briefly, 20 μL of cAMP (8 mM) was added to 20 μL of recipient bacterial suspension in logarithmic phase (at OD_600_ of 1.0) and incubated for 10 min. 1 μg of the recombinant plasmid was added into this bacterial suspension and incubated for another 10 min, which was subsequently spotted on a TSA/V/S plate for 5 h at 37℃. Bacterial cells were finally collected and transferred to the TSA/V/S/Kan^50^ plate and incubated at 37℃ for 48 h. All the natural transformants growing on TSA/V/S/Kan^50^ were PCR identified with primer pairs P5/P6, P9/P10, and P11/P12. For complementation, the *lsgB* gene operon including the promoter and terminator regions was amplified and cloned into the *E. coil – H. parasuis* shuttle vector pSHK3-Gm [2] to generate the complementation plasmid, pSHK3-C*-lsgB*. This plasmid was then introduced into the *ΔlsgB* mutant by electroporation under the condition of 2.5kV voltage, 5ms pulse time and 2mm electrode gap to obtain the complementation strain C-*lsgB*. The bacterial suspension was grown in TSB/V/S at 37°C for 5h with circular agitation and then spread onto TSA/V/S/Kan^50^/Gen^20^ plates at 37°C for 48 h. All electroporation transformants were PCR identified with the primers mentioned above (Table 2 and Figure 2(a)).

**Table 2.** Sequence of the PCR primers used in this study.

Primers	Sequences (5ʹ-3ʹ)^a^	*Tm*
P1 (*lsgB*-up-BamHI-F)	CGCGGATCC*ACCGCTTGT*GGGGTACAAATTGCGAGTAT	60°C
P2 (*lsgB*-up-kan-R)	**TTTTTATCTTGTGCAATG**CTCACTCTGTTTTTTTTCAACTATC	
P3 (*lsgB*-kan-down-F)	**CTCGATGAGTTTTTCTAA**ATTATTTTGTATCGCAGTTCAG	60°C
P4 (*lsgB*-down-PstI-R)	TAACTGCAG*ACAAGCGGT*CAATAATGTCGCAGAATACC	
P5 (Kan-F)	TTAGAAAAACTCATCGAGCATCA	50°C
P6 (Kan-R)	CATTGCACAAGATAAAAATATATCA	
P7 (C*-lsgB*-F-EcoRI)	GGAATTC*ACCGCTTGT*TATTTTTATTGCTGCTCTAGCTTAT	61°C
P8 (C*-lsgB*-R-XbaI)	GCTCTAGA*ACAAGCGGT*CTATTGGCATGTGTAGTCAATTACT	
P9 (Inter-*lsgB*-F)	TAGTATTTTATTCGGTCTGG	45°C
P10 (Inter-*lsgB*-R)	AATTAACTCTTTCTTTGGAG	
P11 (Intact-*lsgB*-F)	GAGATTTTTTATTTACTAGTATTTT	58°C
P12 (Intact-*lsgB*-R)	TACATTAGGTAAATTAATAAAACTA	
GalR1-F	GATACTGTGAGCATGTCATTAAGAG	65°C
GalR1-R	ACTGTAAAGCTCCTCCTTTGTTTAC	
GalR2-F	TATGGACGCGGGGGGC	65°C
GalR2-R	AGCTGTTCGGGTGGCTGTGGCGGTG	

^a^Restriction enzyme sites are underlined, uptake sequences (USS) are in italics, overlapped sequence are in bold.

### Growth analysis of *H. parasuis* wild-type, *ΔlsgB* mutant and C-lsgB strains

To compare the growth of wild-type SH0165, *ΔlsgB* mutant, and C-*lsgB* strains, overnight cultures of each strain were diluted in TSB/V/S and adjusted to the optical density at 600 nm (OD_600_) of 1.0. The cultures were then inoculated at 1:1000 into fresh medium and incubated at 37°C with circular agitation (180 rpm· min^−1^) for 16 h. The OD_600_ of the cultures was measured at 2 h intervals and colony forming units (CFUs) were determined by serial dilution and plating at 6 h intervals. Data collection at each time point was performed three times independently.

### Autoagglutination test

The autoagglutination ability of *H. parasuis* wild-type, *ΔlsgB* mutant and C-*lsgB* strains was evaluated as previously described with slight modifications [22]. Briefly, overnight cultures of each strain were inoculated into 5mL of TSB/V/S medium and cultured at 37°C for 10 h. Bacterial cells were collected and diluted in fresh medium to OD_600_ 0.7, and the mixture was subsequently statically incubated at 25°C. The OD_600_ values of the upper suspensions were measured every 30 min during 6 h of observation.

### Lipooligosaccharide analysis

LOS from the wild-type SH0165, *ΔlsgB* mutant and *C-lsgB* strains was extracted using the phenol-water method as previously described with slight modifications [53]. Briefly, overnight bacterial cells were collected and resuspended in sterile deionized water at a ratio of 10% (g/ml). An equivalent amount of 90% phenol solution was added and the mixture was incubated in a water-bath with agitation at 68°C for 30 min and was then precipitated overnight at 4°C. This operation was repeated 4 times and the supernatant recovered was the LOS crude extract. Next, this crude extract was dialyzed to remove the phenol, and the aqueous phase was further precipitated with 70% ethanol. This precipitate was finally dissolved in the sterile deionized water and used as the LOS refined extracts. LOS preparations were then separated via sodium dodecyl sulfate polyacrylamide gel electrophoresis (SDS-PAGE; 9% stacking gel and 17% separating gel) and visualized via silver staining.

### Sialic acid extraction and high-performance liquid chromatography (HPLC)

Sialic acid content was quantitatively determined using a Sialic Acid Fluorescence Labeling kit according to the manufacturer’s instructions (Takara, Japan). Briefly, sialic acid from the wild-type, *ΔlsgB* mutant and *C-lsgB* strain was extracted with 50 mM HCl at 80°C for 3 hours, and 50 μL of cell-free culture supernatant was then collected by centrifugation and mixed with 200 μL 1,2-diamino-4,5-methyleneoxybenzene (DMB) at 50°C for 2.5 hours. Samples were next applied to a PALPAK Type R column which had been washed with 100% methanol and equilibrated with 30% methanol and a flow rate of 0.9 mL/min. Chromatographic analysis was performed on a Waters 2695 series (USA) instrument and the peak was detected by fluorescence (Ex.373nm, Em.488nm) at appropriate time points according to the standard substance.

### Gram staining and scanning electron microscopy

Morphological observation of the wild-type, *ΔlsgB* mutant and C-*lsgB* strains was performed via Gram staining and scanning electron microscopy (SEM). Gram staining of each strain was performed following standard procedures, and SEM was conducted according to our previously described methods [2].

### Serum resistance assay

The porcine serum used consisted of a pool of sera collected from eleven healthy piglets (5–6 months old) that were free of Glässer’s disease and negative to *H. parasuis* antibodies. Mouse serum was collected from twenty specific pathogen free grade BALB/c mice and pooled. Sera were sterilized using a 0.22 μm filter and aliquots were stored at −80°C until use. Some aliquots were heat-treated at 56°C for 30 min to inactivate the complement components. The serum resistance assay was performed following an established procedure with brief modification [52]. Briefly, 100 μL of bacterial suspension in logarithmic phase (approximately 2 × 10^8^ CFU· mL^−1^) was mixed with either 100 μL of the normal serum (NS) or the heat-treated serum (HS) to obtain a final concentration of 50% serum. The mixture was maintained at 37°C with slight circular agitation (20 rpm· min^−1^) for 1 h. After incubation, the samples were serially diluted 10-fold and spotted onto plates and incubated at 37°C for 24 h and bacterial colonies counted. Results are presented as the percent survival calculated by dividing the number of bacteria treated with normal serum (NS) by the number of bacteria treated with heat-inactivated serum (HS). The assay was performed in triplicate.

### *In vitro* adhesion and invasion assays

Both PK15 and PIEC cells were used to test the adhesion and invasion abilities of the wild-type, *ΔlsgB* mutant, and C-*lsgB* strains as previously described [29,31,54]. For the adhesion assay, bacteria were grown overnight in 5 mL TSB/V/S at 37°C with agitation. Bacterial cells were collected, washed and resuspended in fresh cell culture medium, and added at 2 × 10^7^ CFUs/well onto confluent cell monolayers grown in 24-well plates. The plates were incubated for up to 2 h at 37°C to allow bacterial adhesion. Cells were then washed five times with PBS to eliminate unbound bacteria and treated with 200 μL of 0.25% trypsin/EDTA. Thereafter, 800 μL of prechilled-deionized water was added to lyse the cells, and bacterial counts were determined by plating at appropriate dilutions. In adhesion inhibition tests, the cells were pretreated with D-galactose prior to the addition of bacteria.

For the invasion assay, bacteria were added to cells as described above. Subsequently, cells were washed three times to remove unbound bacteria and then incubated in medium containing 100 U· mL^−1^ penicillin and 10 μg· mL^−1^ streptomycin sulfate for a further 1 h to kill extracellular bacteria. Cells were washed and lysed as described above. The released intracellular bacteria were quantified by plating at appropriate dilutions. Each assay was conducted in triplicate.

### Western blotting, whole cell ELISA, and real-time PCR

Serum factor H (fH) binding to *H. parasuis* was detected by western blotting and by whole cell ELISA. For Western blotting, 4 × 10^8^ CFU bacterial cells resuspended in 180 μL of Hanks’ Balanced Salt Solution (HBSS, Mediatech, Inc., MD, USA) were incubated with 20 μL of normal porcine serum at 37°C for 1 h with moderate agitation. The mixtures were then added to HBSS buffer containing 20% sucrose and centrifuged at 10,000 × g for 3 min to separate the upper free fH from the lower bacteria-bound fH. The pellets were resuspended in 1% SDS and boiling-lysed for immunoblotting analysis with 0.45μm PVDF membrane. Blots were incubated with polyclonal anti-fH antibody (Abcam, 1:500) for 3–5 h at room temperature, and with horseradish peroxidase (HRP)-conjugated anti-goat secondary antibody (Abclonal, 1:5000). Blots were visualized using enhanced chemiluminescence solution (Bio-Rad).

For whole cell ELISA, the assay was performed as previously described with slight modifications [55]. Briefly, 10^8^ CFU of bacteria were incubated with 20 μL of normal porcine serum (60 min at 37°C) as described above for western blotting. Incubation was stopped by washing three times with chilled-HBSS containing 5 mM PMSF in a refrigerated microcentrifuge. The bacterial pellets were resuspended in 200 ml of the same buffer, and 50 μL of each sample post-incubation was added per well of a 96-well U-bottomed polystyrene microtiter plate and coated overnight at 4°C. The plates were then washed with PBS containing 0.05% Tween-20. Primary anti-fH antibody was diluted in PBS as abovementioned in Western blotting (1:500) and incubated at room temperature for 2h, and secondary Cy3-labeled anti-rabbit IgG was diluted in PBS/0.05% Tween-20 (Beyotime Biotechnology, 1:5000) and incubated for another 1h. To normalize the measurement, the result was presented as the average fluorescence of each single bacterium.

For real-time PCR, cells were challenged with the different strains for 2 h as mentioned above. Cells were immediately washed with chilled-PBS, and total RNAs were extracted using Trizol reagent (Invitrogen, Carlsbad, CA, USA) according to the manufacturer’s instructions. cDNAs were synthesized using a PrimeScript™ RT reagent kit with gDNA Eraser (TaKaRa, Japan) according to the manufacturer’s instructions. Real-time PCR was performed with a ViiA™ 7 Real-Time PCR System using Power SYBR Green PCR Master Mix (Applied Biosystems, USA). GAPDH was amplified as an endogenous control and results were normalized to GAPDH and calculated by the 2^−ΔΔ*CT*^ method. The assays were repeated three times to confirm the results. Primers used for real-time PCR are listed in Table 2.

### Flow cytometry analysis

Flow cytometry was applied to compare the deposition of C3b or membrane attack complex (MAC) on wild-type, *ΔlsgB* mutant, and C-*lsgB* strains. In brief, 5 × 10^8^ CFUs of log-phase bacteria were incubated with 250 μL of 50% pig serum at 37°C for 30 min followed by extensive washes and resuspension. For detection of the MAC deposition, bacterial cells were incubated with primary antibody against MAC (Abcam, Cambridge, Massachusetts) at room temperature for 30 min, and then incubated with FITC-conjugated secondary antibody. For detection of the C3b deposition, bacterial suspension was incubated directly with FITC-conjugated anti-C3 antibody (Biolegend, California, USA) without secondary antibody incubation. Bacterial surface deposition of C3b or MAC was analyzed on a FACS Calibur Flow Cytometer (Becton Dickinson, Franklin Lakes, NJ, USA).

### Virulence assays

Animal infection assays were conducted to compare the pathogenicity and the *in vivo* bacterial load of these strains. All procedures and handling techniques conformed to the guidelines established by the China Regulations for the Administration of Affairs Concerning Experimental Animals (1988) and Regulations for the Administration of Affairs Concerning Experimental Animals in Hubei (2005) (Project No.00263863 and Animal Welfare Assurance No.170211). Female BALB/c mice (4 weeks old) were purchased from China Three Gorges University (Hubei, China, Quality Certifcate No.42000600020031). All efforts were made to provide the ethical treatment and minimize suffering of animals employed in this study.

Mice were randomly divided into four groups of 10, and intraperitoneally injected with the different strains at a dose of 1 × 10^9^ CFU per mouse. Clinical symptoms post infection was carefully monitored for 7 days of observation, and survival was recorded every day. In another assay, at 8 h post infection, blood was taken for serum isolation, and mice were then euthanized and perfused. The spleen and lung were harvested, weighed, homogenized, and plated to determine bacterial colony counts.

## Statistical analysis

GraphPad Prism software ver. 6.0 was used for graph plotting and statistical analysis. The data were evaluated by analysis of variance (Two-way ANOVA, Dunnett’s multiple comparisons test) and Student’s *t*-test. A *P* value of < 0.05 was considered to be statistically significant (*), while *p *< 0.01 was considered extremely significant (**).

## Abbreviations

LOS: lipooligosaccharide
NAD: nicotinamide adenine dinucleotide
CMP: Cytidine monophosphate
MP-Neu5Ac: Cytidine monophosphate-N-Acetylneuraminic acid
TSA: tryptic soy agar
TSB: tryptic soy broth
PK15: porcine kidney cells
PIEC: porcine iliac artery endothelial cells
OD_600_: optical density at 600 nm
CFUs: colony forming units
HPLC: high performance liquid chromatography
SEM: scanning electron microscopy
DMB: 1,2-diamino-4,5-methyleneoxybenzene
NS: normal serum
HS: heat-treated serum
fH: factor H
HBSS: Hanks’ Balanced Salt Solution
MAC: membrane attack complex
GalR: galactose binding lectin domain
Kan: kanamycin
Gm: gentamicin
